# Response of the weeping lizard to distress calls: the effect of witnessing predation

**DOI:** 10.1007/s10071-023-01743-8

**Published:** 2023-01-21

**Authors:** Antonieta Labra, Andrea Zapata

**Affiliations:** 1 Centre for Ecological and Evolutionary Synthesis (CEES), Department of Biosciences, University of Oslo, Oslo, Norway; 2 NGO Vida Nativa, Santiago, Chile; 3 Platón 931, Ñuñoa, Santiago, Chile

**Keywords:** Antipredator responses, Immobility, *Liolaemus chiliensis*, Predation, Public information

## Abstract

Escaping from predation saves life, but costs energy and time. The “threat-sensitive predator-avoidance” hypothesis proposes that prey may modulate their antipredator responses, and thus the associated costs, in accordance with the magnitude of predation risk. This process requires that prey accurately assess this risk by decoding available information from various sources. For example, distress calls are uttered by prey when a predator traps them and can serve as public information on predation risk. Such is the case for the weeping lizard whose distress calls trigger immobility in conspecifics. Here, we tested whether this antipredator response of the weeping lizard is modulated by witnessing predation. We exposed lizards to distress calls alone or paired with models of a prey (conspecific), a predator (snake), or a predatory event (a snake subjugating the conspecific). Data show that the sole presence of the predator or prey paired with distress calls seems not to modulate the antipredator responses. Contrarily, witnessing a predatory event associated with calls intensified antipredator responses; lizards reduced their activity for longer and avoided proximity to the stimuli, which may decrease predation risk by reducing the likelihood of being detected by the predator. We conclude that the weeping lizard can use multisensorial public information to assess predation risk and modulate its antipredator responses.

**Supplementary Information:**

The online version contains supplementary material available at 10.1007/s10071-023-01743-8.

## Introduction

Avoiding being eaten is one of the biggest challenges for most animal species across their life span, which makes predation a selective pressure that modulates the evolution of different prey characteristics (e.g., morphology, behavior, life history traits; Kavaliers and Choleris 2001; Ruxton et al. 2004; Caro 2005). In the short term, escaping predation has associated costs, such as the energy used to run away (Jermacz et al. 2022) or to fight against the predator (Feder and Arnold 1982; Crofoot 2012). Moreover, antipredator responses (e.g., remaining hidden; Martín and López 1999; Jennions et al. 2003) divert time from fitness-enhancing activities such as food search (Eifler et al. 2008), food consumption (Curé et al. 2015; Catano et al. 2016), or thermoregulation (Stapley 2004). The “threat-sensitive predator-avoidance” hypothesis proposes that prey may alter their responses according to the magnitude of predation risk (Helfman 1989). For this, it is pivotal that prey have an accurate and reliable risk assessment to adjust the time and/or energy allocated to antipredator response (Lima and Dill 1990; Lima and Bednekoff 1999; Kavaliers and Choleris 2001; Stankowich and Blumstein 2005). Several studies support this hypothesis revealing the cues used by prey in risk assessment and how these modulate their antipredator responses. For example, the wolf spider *Pardosa milvina* evaluates the size of predators using their scents (i.e., chemical cues), displaying more intense reactions toward larger predators (Persons and Rypstra 2001). Similarly, the lizard *Ctenosaura similis* modulates its escape responses according to the eye size of the predator (Burger et al. 1991).

Predation risk not only can be assessed by direct experience, but also by using public information provided by other prey, a relatively cost-free assessment (Lima and Steury 2005). For example, many social bird and mammal species emit calls when detecting a predator (i.e., alarm calls; for reviews, see Klump and Shalter 1984; Zuberbühler 2009). Conspecifics may react to these calls with antipredator responses, such as the increased vigilance showed by the chipmunk *Tamias striatus* (Baack and Switzer 2000) or the longer inactive period exhibited by the warbler *Dendroica petechia* (Gill and Sealy 2003). In addition, responses can be accurate because calls can provide information, for example, on the type of predator (e.g., aerial, terrestrial; Rendall et al. 2009; Suzuki 2015; Diggins 2021) or risk level (e.g., predator proximity; Dutour et al. 2021; Elgar and Riehl 2021). Moreover, because call emission usually is accompanied by information from other sensory channels (e.g., prey visual displays; McRae and Green 2014), multisensory information may improve risk assessment (Kavaliers and Choleris 2001; Lima and Steury 2005; Munoz and Blumstein 2012). Such is the case for the squirrel *Sciurus carolinensis*, which intensifies its antipredator responses when observing a conspecific flapping its tail (alarm display) while simultaneously uttering alarm calls (Partan et al. 2009).

Different tetrapod species also emit distress calls, i.e., vocalizations uttered by prey when they are cornered, attacked, or trapped by a predator (Klump and Shalter 1984; Magrath et al. 2015). These calls can have different functions, such as scaring the primary predator or attracting a secondary one (Perrone 1980; Högstedt 1983), and also can be public information on predation risk to which conspecifics may react with antipredator responses (Conover and Perito 1981; Hoare and Labra 2013). The primary visual information associated with such calls is the predatory event, i.e., a predator cornering, trapping, killing, or consuming prey. Conover and Perito (1981) showed that starlings (*Sturnus vulgaris*) took longer to return to a foraging area where they experienced distress calls paired with the predator's presence, proposing that a co-presentation of calls with a predator holding prey might induce more intense antipredator responses (see also Conover 1994). Data by Peterson and Colwell (2014) partially support this hypothesis, showing that corvids significantly avoided areas where they found a predator (human) that, after subduing a conspecific (model), left the area, leaving the “dead” conspecific while distress calls were reproduced.

*Liolaemus chiliensis*, the weeping lizard, only vocalizes when it is trapped by a predator (i.e., distress calls; Labra et al. 2013). Conspecifics react to these calls with immobility (Hoare and Labra 2013; Labra et al. 2016; Ruiz-Monachesi and Labra 2020), a behavior that may enhance the probability of remaining undetected by a visual predator (Cooper and Blumstein 2015). In the present study, we test whether witnessing the predatory event modulates the antipredator response of the weeping lizard to distress calls. In the context of the “threat-sensitive predator-avoidance” hypothesis (Helfman 1989), we postulate following Conover and Perito (1981) that the stimuli co-occurrence (i.e., distress calls + predatory event) is perceived as riskier, triggering more intense antipredator responses, i.e., more prolonged immobility, than when calls are perceived alone.

## Methods

During the austral spring (September–October) of 2010, we collected 22 weeping lizards (9 ♀, 13 ♂; mean snout–vent length—mm 89.44 ± 1.60 SE) at Melipilla (33°41’S, 71°13’W; Chile). As in Fong et al. (2021), we transported lizards to the laboratory, where they were maintained individually in plastic enclosures (44.5 × 32 × 25 cm) in an isolated room with temperatures ranging between 30 °C and 12 °C associated with a 13:11 L:D photoperiod. Enclosures had an inverted tile used as a basking place and shelter, a pot to keep a constant water supply, a wooden stick used as perch, and a pot with grass for environmental enrichment; the floor had a sand layer of 3 cm. Lizards received food (*Tenebrio mollitor* larvae) dusted with vitamins three times per week. The week before the experiments, individuals remained undisturbed except for feeding, and at the end of the experiments, lizards were returned to their georeferenced collecting locations.

### Experimental design

**Fig. 1:**
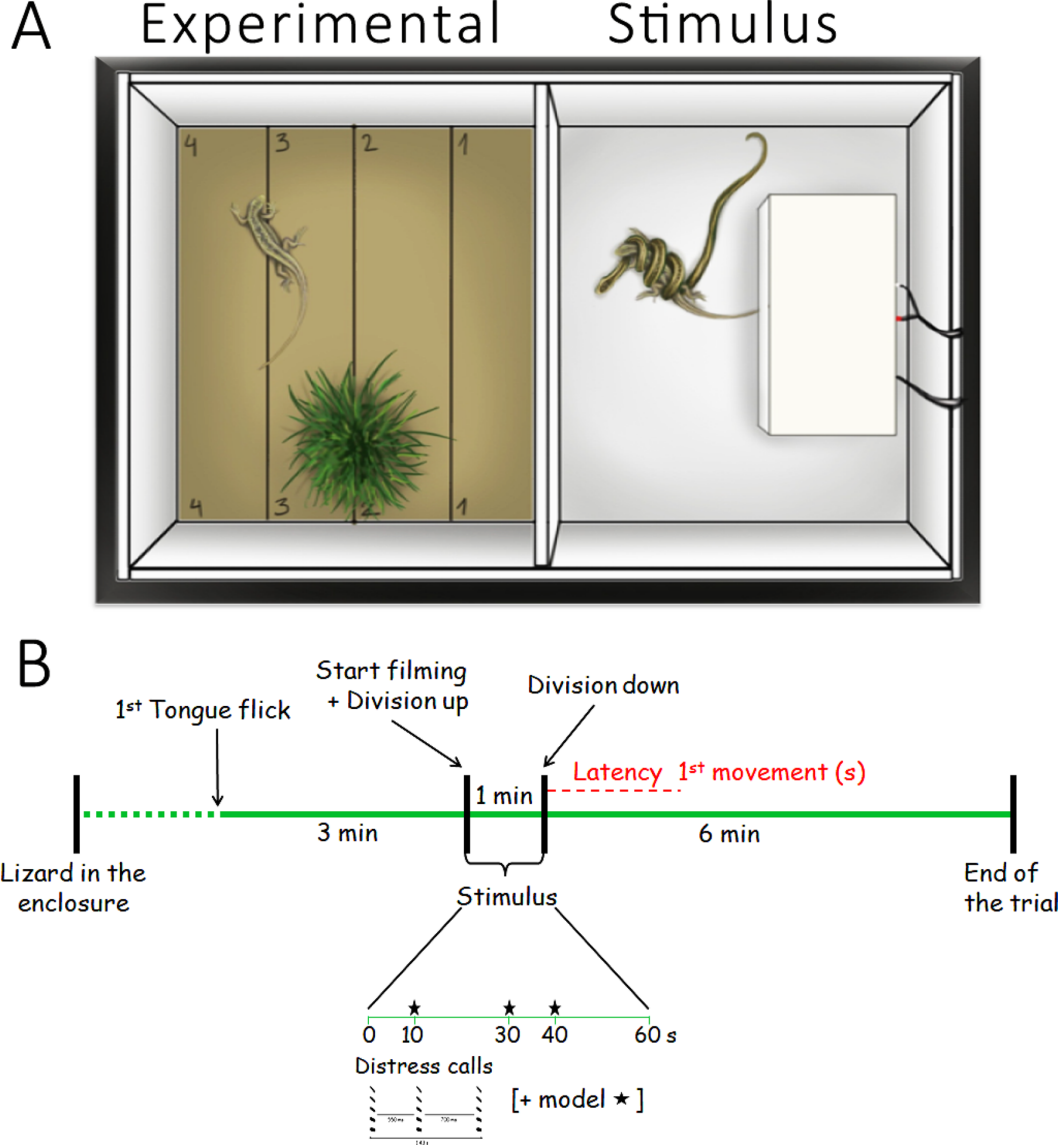
Experimental setup. **A** The experimental enclosure had two areas: experimental and stimulus. The illustration shows the predation treatment (distress call + predatory event), a snake model subjugating a weeping lizard model in the stimulus area. Behind the models is the loudspeaker covered by a white cloth. The floor had brown rugs (not shown in the stimulus area), and the rug of the experimental area had divisions. Figure courtesy of J. Constanzo-Chávez. **B** Representation of the experimental design showing its different stages. The vertical lines indicate the events in the trial, and the dashed lines represent the variable recording time. Below the stimulus period is shown a sonogram with the distress calls, and when the acoustic stimulus was played back, 10 s from division lift. Asterisks in the timeline indicate when models were manipulated

In an acoustically isolated room, we performed experiments using an acrylic enclosure (80 × 40 × 40 cm) divided into two halves (40 × 40 cm each) by a removable opaque plate. We placed the focal lizard in one section (henceforth, experimental area), which had on the floor a thin brown rug with divisions (Fig. 1A) that allowed testing whether lizards show zone preference or avoidance; each lizard had its own rug. We also placed the pot with grasses from the maintenance enclosure to provide a familiar element (Fig. 1A). Finally, using infrared lamps, we maintained the experimental area at 35 °C, the species’ selected body temperature (Labra et al. 2009).

The other section housed the stimuli (henceforth stimulus area; Fig. 1A). The floor also had a brown rug, and we placed a loudspeaker (Behringer^®^) covered by a thin cloth, connected to an amplifier (NAD Electronics 3020i), and this to an iPod nano A1320 to reproduce the distress calls that were set at 51 dB RMS SPL at the center of the experimental area. In addition to the distress calls, we used three-dimensional silicone models of a lizard and a snake, with colors and dorsal patterns mimicking, respectively, a weeping lizard and a *Philodryas chamissonis*, a lizard predator (Greene and Jaksić 1992).

For the experiments, we took the focal lizard from its maintenance enclosure and placed it in a cloth bag for 10 min (each lizard had its own bag) to reduce handling-associated stress (Labra 2011). Then, on the floor of the experimental area, we allowed the lizard to exit freely from the bag, after which we removed the bag, and the researcher left the area to remain behind a wall during the trial. At 130 cm above the experimental area, a camcorder connected to a TV monitor allowed us to follow the lizard’s behavior and determine when it made its first tongue flick (Fig. 1B), the onset of the chemical exploration (Labra 2011) and the trial; if no tongue flicks had occurred after 10 min, we canceled the trial. After this first tongue flick, the lizard had 3 min to explore the section, and then we started the recording (Fig. 1B). We lifted the division and maintained it up for 1 min to present the stimuli, ending the stimulus period by lowering the division. Finally, we kept recording for 6 min (Fig. 1B), a period when corticosterone may be high (Trompeter and Langkilde 2011), which might enhance antipredator responses (Thaker et al. 2009). At the end of the trial, we removed the lizard and measured its cloacal temperature to ensure that this was around the species' selected body temperature, 35 ± 2 ℃ (Labra et al. 2009), to avoid behavioral differences due to variation in body temperature (Shine et al. 2000). We placed the lizard and the pot with grasses back in the maintenance enclosure. The lizard had an inter-trial resting period of at least 2 days.

Because the weeping lizard responds to conspecific scents (Labra and Hoare 2015; Valdecantos et al. 2020), we cleaned the experimental area and the division plate with alcohol to remove potential chemical traces left by the lizard and changed the rug to avoid affecting the behavior of the next lizard. We used disposable gloves during the whole procedure, replacing them between trials.

### Treatments

To control for the effects of variation in the distress calls (Gerhardt 1992), we used a synthetic call previously produced and used by Hoare and Labra (2013). Briefly, this call was the average of distress calls emitted by 13 individuals. It had a downward frequency-modulated pattern, five harmonics, and a duration of 60 ms. The whole acoustic stimulus was composed of three identical distress calls separated by two silence intervals (550 and 700 ms; Fig. 1B).

We manipulated the models with transparent threads to simulate “life” and create a more realistic situation relevant to modulate prey responses (Carlson et al. 2017). Movements were standardized across the trials, although slight variations helped minimize pseudoreplication. Models were moved once at 10, 30, and 40 s after lifting the division (Fig. 1B).

All the lizards were exposed to the following four treatments in a counterbalanced design to avoid an effect of treatment order:

1. Distress call (DC; henceforth call). We reproduced only once the complete acoustic stimuli 10 s after lifting the division (Fig. 1B), and 40 s later, we lowered the division. This protocol was the same for all treatments.
2. Lizard + DC (henceforth prey). The lizard model was alone in the stimulus area, and its snout pointed to the experimental area. The upper body segment was lifted up and back, mimicking the lizard behavior when distress calls are uttered (A. Labra, pers. obs.). In preliminary tests, lizards exposed to this model tended to approach it and eventually performed head bobs, suggesting that lizards recognized the model as conspecific.
3. Snake + DC (henceforth predator). The snake model was alone in the stimulus area. Its head was the only mobile part lifted and moved laterally in zone 1. Previously, Constanzo-Chávez et al. (2018) showed that lizards respond to this model with antipredator behaviors (i.e., escapes), indicating that lizards recognized this model as a predator.
4. Predation event + DC (henceforth predation). The snake was “subjugating” the lizard (Fig. 1A), and the movement of the set (snake + lizard) simulated a small jump such as those observed when a snake fights with prey that attempts to escape, while constricting it.

The prey and predator treatments allowed exploring whether the presence of each participant in a predatory event alone would have a similar effect as the predator–prey interaction (e.g., Conover and Perito 1981). A treatment with just the movement of the division was not included, as any effect it might cause on the lizards’ behavior would be the same across the treatments.

### Recorded variables

As Valdecantos et al. (2020) pointed out, the weeping lizard usually exhibits a limited behavioral repertoire, and in this study, six behaviors were recorded from the videos, defined in Table 1. The Movement variable included the time that lizards exhibited defecation as well as cloaca and snout dragging (Table 1). These three behaviors occurred at a very low frequency to be analyzed separately, which in total were observed on seven occasions. The only other behavior exhibited by lizards was eye-bulging (i.e., eye protrusions; Reyes-Olivares et al. 2016), which was not included in the Movement variable, as the behavior only involves a slow eye protrusion and retraction. This behavior was exhibited only once by trial and occurred in 16 out of the 88 trials, equally distributed across treatments and, therefore, it was not analyzed. During the stimulus presentation, lizards mostly remained immobile, and we did not consider this period. For consistency, a single researcher (AZ) scored the videos.

**Table 1:** Behavior recorded from videotapes for *Liolaemus chiliensis* after the presentation of distress calls alone or paired with models (prey, predator, or predatory event)

Variable	Definition	Reference
Latency to the first movement (s)	The period between the end of the stimulus and the onset of any behavior (e.g., tongue flick, displacement, scanning). See Fig. 1	(Hoare and Labra 2013; Fong et al. 2021)
Scanning (s)	The lizard remains quiet, but makes slight head/eye movements, particularly toward the location of the stimulus	(Trompeter and Langkilde 2011; Hoare and Labra 2013)
Movement (s)	The total time the lizard moves, including displacements, escape attempts and position adjustments without changing place. This variable also included the time spent performing behaviors that occurred in low frequency: defecation and cloaca or snout dragging	(Hoare and Labra 2013; Fong et al. 2021)
No. of tongue flicks	Number of times that lizard protruded their tongue, directed to the air or a surface	(Fong et al. 2021)
Time in a section (1 or 4; s)	The time that the lizard spent in specific zones of the experimental area, close and far from the stimulus, 1 or 4, respectively (see Fig. 1A). When a lizard was in two zones, we considered the one where the head was	Present study

### Statistical analyses

We used repeated-measures analysis of generalized linear models (GLM) to determine the treatment effect on the antipredator responses of the weeping lizard, followed by Fisher LSD tests. The normality of some variables was improved by transforming them (see Table 2). Analyses were performed using Statistica 7.0 (StatSoft, Inc., 2002, Tulsa, OK, USA).

**Table 2:** Repeated-measures analysis of generalized linear models, with statistically significant values in bold

Variable	GLM	
	*F*	*p*
Latency to the first movement	3.100	**0.033**
Scanning	2.899	**0.042**
Movement	3.493	**0.021**
No. of tongue flicks	1.980	0.126
Time in section 1	3.631	**0.018**
Time in section 4	1.219	0.310

Variables scanning, movement, and time in section 1 were log_10_ transformed

In all cases, the degrees of freedom were 3 and 63, and the sample size was 22

## Results

**Fig. 2:**
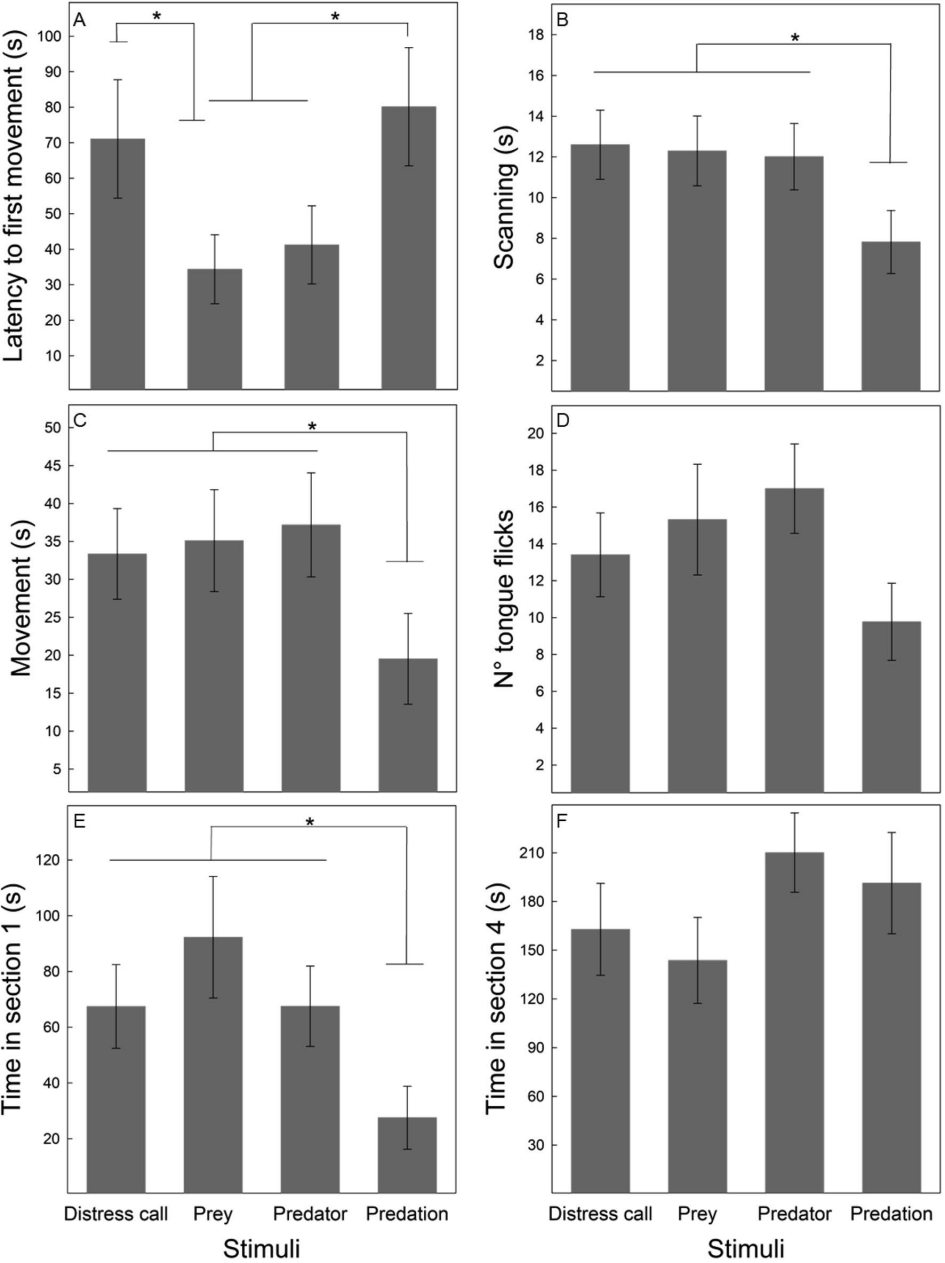
Mean untransformed data (± SE) of six responses recorded in *Liolaemus chiliensis* after the exposure to four stimuli, which combined acoustic (distress calls) and visual information (reptile models) involved in a predatory event: distress calls (DC) of a conspecific, prey (lizard + DC), predator (snake + DC), and predation (predatory event, the snake subjugating the lizard + DC). * = *p* < 0.05. **A** Latency to first movement, **B** Scanning (s), **C** Movement (s), **D** N° tongue flicks, **E** Time in section 1 (s) and **F** Time in section 4 (s)

Table 2 shows the GLM results of each variable; the Latency to the first movement after the stimulus was longer in the call and predation treatments than in the others, except for the call and predator treatments, which were similar (Fig. 2A). After lizards restarted their activity, they scanned (Fig. 2B), and moved less (Fig. 2C) as well as spent less time in the section closest to the stimulus, section 1 (Fig. 2E), when they had been exposed to the predation treatment as compared to the other treatments. The reduced activity (movement and scanning) might result from less available time due to the long latency. Therefore, we reanalyzed these variables, adjusting them by the available time after discounting the latency period. Results showed the same trends; significant differences among treatments (both variables log-transformed, scanning: *F*_3,63_ = 2.92, *p* = 0.041; movement: *F*_3,63_ = 2.96, *p* = 0.039), due to the least activity exhibited in the predation treatment. Finally, the number of tongue flicks and time spent in section 4 (Fig. 2D and F) were similar across treatments.

## Discussion

Previous studies have shown that weeping lizards respond to distress calls with immobility (Hoare and Labra 2013; Labra et al. 2016; Ruiz-Monachesi and Labra 2020). Here, we found that lizards exhibited an extended activity reduction when witnessing the predatory event paired with calls, suggesting that the co-occurrence of public information was perceived as high risk. Contrarily, the sole presence of predator or prey did not modify the antipredator responses to calls, except by the latency to the first movement.

Witnessing predation did not modulate the restoration of the activity after the stimulus presentation (i.e., latency to the first movement), as it was similar to when calls were played back alone. The shorter latency exhibited by lizards when exposed to prey and predator treatments suggests that these experimental conditions were perceived as low predation risk. We hypothesize that because the weeping lizard only vocalizes when it is trapped by a predator (Labra et al. 2013; Constanzo-Chávez et al. 2018), the co-presentation of distress calls and the predator or prey alone would be an incongruent stimuli interaction (see below for further discussion), triggering an earlier reactivation. However, after lizards resume their activity in the prey and predator treatments, they behave similarly to when distress calls are presented alone.

Once lizards restarted their activity, witnessing the predatory event paired with distress calls triggered a reduction in activity and an avoidance of the danger zone close to the predatory stimuli. However, the safest area, farthest from the predatory event, was not preferred. Avoiding proximity to the predatory event suggests that the lizard that witnessed the event might perceive a risk in that the captured prey may escape and make the observer the predator’s next target. On the other hand, because prey detection usually depends on the target movement (Cooper and Blumstein 2015), including in snakes (e.g., Burghardt and Denny 1983; Licht 1986), the reduced activity after witnessing predation may enhance the lizard’s chances of remaining undetected by the predator. Moreover, some lizard species even reduce tongue flicking when snake scents are perceived (Labra and Niemeyer 2004).

The sole presence of the snake or conspecific did not modulate reactions to distress calls, contrasting with responses observed in other taxa; e.g., although gulls and crows show more intense responses when the predator has captured a prey, they also react to the presence of predators or dead conspecifics presented alone (Kruuk 1976; Swift and Marzluff 2015). Similarly, some bird species exposed to distress calls paired with predator presence (*Sturnus vulgaris*, Conover and Perito 1981; *Parus bicolor*, Hill 1986) or dead conspecifics, in the case of the bat *Myotis lucifugus* (Fenton et al. 1976), intensified the antipredator responses compared to just the calls’ presentation. Differential responses across species may result from different tendencies to aggregate. The mentioned bird and bat species tend to aggregate (Thomas et al. 1979; Pravosudova and Grubb 2000; Zoratto et al. 2010), and with many conspecifics in the surroundings, distress calls in the background may involve an unwitnessed predator-conspecific interaction. In this scenario, the sole presence of a conspecific or a predator paired with calls is not necessarily incongruent information on predation risk. In contrast, the weeping lizard does not aggregate, as reported for many other lizard species (Gardner et al. 2016). Occasionally, two or three individuals may be found close to each other (< 3 m; A. Labra, pers. obs.), and in this scenario, distress calls in the background could only involve a close neighbor, which in the present study was closer than 80 cm (i.e., loudspeaker). At this distance, the predation event could be witnessed and would be the only possible visual public information associated with calls. In this context, lizards confronted with incongruent stimuli, such as the predator or prey paired with calls, seem to react only to the information provided by the distress calls.

Finally, lizards displayed a similar number of tongue flicks across treatments, suggesting that they did not receive chemical stimuli that triggered an exploratory behavior with the tongue; models did not provide new scents (e.g., specific snake or conspecific scents), and the experimental area only had the focal lizard scents (i.e., rug, pot with grasses). We cannot, however, rule out that the visual and acoustic stimuli may provide sufficient information on predation risk, making it unnecessary to search for more information.

We conclude that the antipredator responses of the weeping lizard to distress calls are modulated by witnessing the predatory event, as can be predicted from the **“**threat-sensitive predator-avoidance” hypothesis (Helfman 1989). In addition, the exhibited antipredator responses by lizards when confronting incongruent stimuli, i.e., snake or lizard alone paired with distress calls, seem to be mainly modulated by the distress calls. New studies can unravel whether stimuli interaction (i.e., predatory event and distress calls) provokes an enhancement or modulation of the antipredator responses, when clarifying whether these stimuli are or are not redundant (Partan and Marler 2005; Munoz and Blumstein 2012).

## Supplementary Information

Below is the link to the electronic supplementary material.

Supplementary file1 (XLSX 17 KB)

## Data Availability

All data are available in the supplementary material.
